# MFN2 Overexpression Attenuates Coal Dust-Induced Pulmonary Fibrosis by Modulating MAMs Integrity and Cell Apoptosis

**DOI:** 10.3390/toxics14050391

**Published:** 2026-04-30

**Authors:** Na Zhang, Lulu Liu, Junrong Chen, Yingjie Liu, Shen Yang, Mei Zhang, Yu Xiong, Xin Ma, Yan Wang, Xiaoqiang Han

**Affiliations:** 1 School of Public Health, Ningxia Medical University, No. 1160, Shengli Street, Xingqing District, Yinchuan 750004, China; 230230020193@nxmu.edu.cn (L.L.); cjr250240510183@nxmu.edu.cn (J.C.); 1143295659@nxmu.edu.cn (Y.L.); 250230020222@nxmu.edu.cn (S.Y.); 230240210124@nxmu.edu.cn (M.Z.); xiongyu@nxmu.edu.cn (Y.X.); mx2024@nxmu.edu.cn (X.M.); 20180019@nxmu.edu.cn (Y.W.); 2 Ningxia Key Laboratory of Environmental Factors and Chronic Disease Control, No. 1160, Shengli Street, Xingqing District, Yinchuan 750004, China; 3 School of Basic Medical Sciences, Ningxia Medical University, No. 1160, Shengli Street, Xingqing District, Yinchuan 750004, China; hanxqjc@nxmu.edu.cn

**Keywords:** apoptosis, Ca^2+^, coal workers’ pneumoconiosis (CWP), mitochondria-associated endoplasmic reticulum membranes (MAMs), MFN2

## Abstract

Pneumoconiosis, characterized by progressive pulmonary fibrosis, remains a predominant occupational disease in China, with coal workers’ pneumoconiosis (CWP) and silicosis being the primary subtypes. Despite extensive research, its underlying pathogenic mechanisms are not yet fully understood. Mitochondria-associated endoplasmic reticulum (ER) membranes (MAMs) are crucial subcellular microdomains that govern Ca^2+^ transport, sustain cellular bioenergetics, and maintain systemic homeostasis. Emerging evidence has linked the structural and functional dysregulation of MAMs to the pathogenesis of various fibrotic disorders. Apoptosis, a highly regulated cell death process, is a key driver in pneumoconiosis progression, in which Ca^2+^ imbalance serves as a critical signaling cascade. Mitofusin 2 (MFN2), a core regulator of MAMs’ structural integrity, mediates mitochondrial fusion and directly bridges the ER with the outer mitochondrial membrane, thereby stabilizing ER–mitochondrial coupling. However, whether MFN2 mitigates fibrosis by preserving MAMs’ integrity and subsequently suppressing Ca^2+^-dependent apoptosis remains elusive. In this study, we established SD rat and A549 cell models of CWP. Our results demonstrated that MFN2 expression was downregulated after coal dust exposure, accompanied by MAMs impairment, Ca^2+^ imbalance, and increased apoptosis, which ultimately drove the pathological progression of pulmonary fibrosis. Notably, MFN2 overexpression restored MAMs’ structure and Ca^2+^ homeostasis, alleviated abnormal apoptosis, and subsequently inhibited fibrosis. This study highlights the importance of the MFN2–MAMs–Ca^2+^–apoptosis axis and identifies MFN2 as a potential therapeutic target for pneumoconiosis.

## 1. Introduction

Coal dust is primarily composed of fixed carbon, supplemented by inorganic components such as free silica, alumina, and iron oxide, as well as trace amounts of sulfur, nitrogen, and hydrogen. It exhibits high chemical stability, low flammability and explosivity, and weak adsorption capacity. Respirable coal dust particles containing free silica can deposit in the alveoli, inducing chronic pulmonary inflammation and progressive pulmonary fibrosis, which underpins the pathogenesis of coal workers’ pneumoconiosis (CWP). CWP is a legally defined occupational disease that is highly prevalent in the Chinese coal industry, especially among workers in small-scale and medium-sized coal mines. The risk is closely related to the duration of dust exposure, representing a severe threat to the occupational health of coal workers [1,2,3]. The primary pathological feature of CWP is irreversible pulmonary fibrosis caused by the accumulation of coal dust, which leads to the destruction of alveolar architecture and excessive abnormal collagen deposition. This pathological condition may later progress into massive fibrosis, leading to dyspnea and eventually respiratory failure. Current clinical treatments are mainly symptomatic, including glucocorticoids, pirfenidone, and other antifibrotic agents. Non-pharmacological interventions include pulmonary rehabilitation and bronchoalveolar lavage. Although these therapeutic strategies can delay disease progression, they cannot reverse established pulmonary fibrosis lesions. Consequently, the prognosis remains poor, and the economic burden is substantial [4,5]. Oxidative stress, inflammatory cascades, and TGF-β1-mediated fibrosis are known to be involved in the pathogenesis of CWP [6]. However, the underlying molecular mechanisms of CWP remain incompletely understood and warrant further investigation.

As previous research reveals, dysregulated apoptosis is a key mechanism promoting the development of fibrotic lesions. In idiopathic pulmonary fibrosis (IPF), the apoptosis rate of alveolar and bronchial epithelial cells is significantly increased, characterized by the upregulation of the pro-apoptotic protein Bax and the downregulation of the anti-apoptotic protein Bcl-2. This imbalance directly disrupts pulmonary homeostasis and accelerates collagen deposition [7]. In liver fibrosis, hepatocyte apoptosis triggers inflammatory cascades to promote fibrosis, whereas hepatic stellate cell (HSC) apoptosis inhibits extracellular matrix (ECM) secretion and serves as a critical target for reversing fibrosis. Renal fibrosis is closely associated with abnormal apoptosis of renal tubular epithelial cells, mitochondrial tRNA alterations, and aberrant activation of the miR-27b-3p/p53 pathways, which induce apoptosis and TGF-β1 release to accelerate fibrosis [8]. In myocardial fibrosis, JNK/p53 pathway activation and angiotensin II-induced endothelial cell apoptosis also contribute to abnormal collagen deposition. Recently, research into the mechanisms of apoptosis has progressed rapidly. Mechanistically, ubiquitin-specific protease 14 (USP14) and O-glucosylation regulate apoptosis via mitophagy. Novel apoptotic interventions include SMAC mimetics combined with histone deacetylase inhibitors and traditional Chinese medicine compounds targeting the Survivin–Caspase pathway [9]. Nonetheless, these advancements have not yet been successfully applied to CWP therapy.

Mitochondria-associated endoplasmic reticulum membranes (MAMs) are critical subcellular structures that regulate intracellular calcium homeostasis, lipid metabolism, and apoptotic signaling. The dysfunction of MAMs is closely associated with the pathogenesis of multiple fibrotic diseases and has become a major research focus in recent years [10,11]. Upregulation of MAMs-localized regulatory proteins, such as the IP3R–Grp75–VDAC1 complex, can trigger calcium signaling disorders, forming a vicious cycle of MAMs dysfunction, apoptotic dysregulation, and fibrosis progression. In liver fibrosis models, HSC activation is accompanied by MAMs structural remodeling and significantly increased expression of MAMs-related proteins, including Fis1 and BAP31. By regulating calcium flux to remodel mitochondrial function, these changes inhibit HSC apoptosis, sustain the activated phenotype, and promote massive ECM secretion. Targeted inhibition of MAMs calcium channels can induce activated HSC apoptosis and markedly alleviate liver fibrosis [12]. In renal fibrosis, MAMs dysfunction in renal tubular epithelial cells is a key inducer of epithelial–mesenchymal transition (EMT). Abnormal MAMs-mediated calcium signals may activate downstream NF-κB and TGF-β/Smad pathways, which promote epithelial cell apoptosis and transdifferentiation into myofibroblasts, thereby accelerating renal interstitial fibrosis [13]. In myocardial fibrosis, MAMs’ structural alterations in cardiomyocytes and fibroblasts modulate lesion formation. Disrupted calcium transport in cardiomyocyte MAMs causes mitochondrial damage and cell apoptosis, followed by inflammatory responses induced by DAMPs. Meanwhile, fibroblast MAMs promote myocardial interstitial fibrosis through calcium-dependent collagen synthesis [14].

Mitofusin 2 (MFN2) is a key molecule that regulates MAMs structure and function, with its expression and activity directly determining MAMs integrity and physiological homeostasis. MFN2 is localized on both the mitochondrial outer membrane and the endoplasmic reticulum (ER) membrane. It functions as a homodimer or heterodimer, acting as a molecular bridge that stably tethers mitochondria and the endoplasmic reticulum. This connection maintains the appropriate spatial distance and structural integrity of the MAMs domain, facilitating efficient material exchange and signal transmission between these organelles [15]. Functionally, MFN2-mediated MAMs stability is essential for maintaining intracellular calcium homeostasis. MAMs represent the primary site for calcium transfer from the endoplasmic reticulum to mitochondria. MFN2 directly modulates calcium flux by regulating calcium transport complexes such as IP3R–Grp75–VDAC1, thereby maintaining mitochondrial calcium homeostasis and supporting normal mitochondrial oxidative phosphorylation. Loss of MFN2 expression or function causes MAMs structural dissociation, leading to impaired mitochondrial calcium uptake, calcium overload, excessive activation of the mitochondrial apoptotic pathway, and enhanced oxidative stress [16]. MFN2 is abundantly expressed in lung tissue, and its expression level is closely associated with lung cell repair and the production of fibrosis-related mediators. Previous studies have demonstrated that MFN2 downregulation contributes to the pathogenesis and progression of idiopathic pulmonary fibrosis [17]. Furthermore, enhanced MFN2 expression promotes mitophagy, thereby attenuating the progression of pulmonary fibrosis [18].

Disruption of intracellular calcium homeostasis, a critical consequence of impaired MAMs structure and function, plays a key role in fibrogenesis and is closely linked to cellular apoptosis. Excessive cytoplasmic Ca^2+^ accumulation activates caspases, modulates Bcl-2 family protein expression, and triggers apoptosis. In contrast, insufficient mitochondrial Ca^2+^ uptake impairs mitochondrial function, further promoting the release of apoptotic factors [19]. In pneumoconiosis, lung parenchymal cells are stimulated by dust particles, leading to disturbed MAMs homeostasis. Downregulation of MFN2 directly damages MAMs’ structure, disrupts Ca^2+^ transport into mitochondria, and destabilizes intracellular calcium balance. Moreover, structural dissociation of MAMs exacerbates dust-induced injury, inflammatory factor release, fibroblast activation, and pulmonary fibrosis. It also induces abnormal apoptosis of lung cells via calcium dysregulation, thereby aggravating lung injury. Conversely, maintaining or restoring MFN2-mediated MAMs function inhibits fibrosis, rescues Ca^2+^ transport, and regulates apoptosis to alleviate lung damage.

Nevertheless, whether MFN2 alleviates coal dust-induced pulmonary fibrosis by regulating MAMs’ structural integrity and modulating apoptosis remains unclear. Accordingly, this study aims to investigate the regulatory role of MFN2 in MAMs-mediated signaling, specifically focusing on (1) the correlation between MFN2 expression and MAMs integrity in CWP models; and (2) the mechanism by which MFN2 preserves MAMs structure and inhibits apoptosis to attenuate fibrosis. By exploring these molecular mechanisms at both cellular and animal levels, we aim to provide a mechanistic foundation for targeting MFN2 as a potential therapeutic strategy for CWP.

## 2. Materials and Methods

### 2.1. Reagents and Antibodies

Trypsin–EDTA (0.25%) and penicillin–streptomycin were obtained from Beijing Solarbio Science & Technology Co., Ltd. (Beijing, China). Dulbecco’s modified Eagle medium (DMEM), fetal bovine serum (FBS), and phosphate-buffered saline (PBS) were purchased from Wuhan Servicebio Technology Co., Ltd. (Wuhan, China). The protein extraction kit, BCA protein assay kit, and high-sensitivity ECL chemiluminescence detection kit were purchased from KeyGEN BioTECH (Nanjing, China). PAGE gel fast preparation kits and protein molecular weight markers were from Shanghai YaMei Biomedical Technology Co., Ltd. (Shanghai, China).

Primary antibodies against α-SMA, Vimentin, MFN2, and GRP75 were purchased from Abcam (Cambridge, UK). Antibodies against VDAC and IP3R were obtained from Thermo Fisher Scientific (Waltham, MA, USA). Antibodies targeting Bcl-2, Bax, Caspase- 3, and E-cadherin were provided by ABclonal Technology (Wuhan, China). GAPDH primary antibody was purchased from Affinity Biosciences (Cincinnati, OH, USA). The secondary antibody, HRP-conjugated goat anti-rabbit IgG, was purchased from UElandy Inc. (Suzhou, China).

### 2.2. Animals and Experimental Design

The Experimental Animal Ethics and Welfare Committee of Ningxia Medical University reviewed and validated the protocol for the use of animals involved in this study (Approval No.: IACUC-NYLAC-2024-046, date of approval: 18 November 2024). Throughout the study, the 3Rs principles (Replacement, Reduction, and Refinement) were strictly followed to minimize animal distress. Mature male Sprague-Dawley (SD) rats (aged 6–8 weeks, weighing 200–250 g) were bought from the Experimental Animal Center of Ningxia Medical University. SD rats were housed under a 12 h light/dark cycle at a controlled temperature of 25 ± 2 °C and humidity of 50–60%, with ad libitum access to food and water.

Following a one-week acclimatization period, the CWP model was established. Rats were anesthetized via diethyl ether inhalation until the loss of corneal and pedal withdrawal reflexes was observed. For modeling, a non-invasive intratracheal instillation was performed: rats were secured in a supine position, the glottis was visualized by gently retracting the tongue, and 1 mL of sterilized coal dust suspension (50 mg/mL) was slowly administered via tracheal instillation. The coal dust used in this study had a particle size of <10 μm and was sterilized by autoclaving to ensure the absence of pathogens. After instillation, the rats were held upright for 1 min with gentle thoracic rotation to ensure uniform dust distribution in the lungs. Control rats received an equal volume of sterile saline (Servicebio, Wuhan, China). Post-surgery, rats were kept warm and monitored until awakening and were monitored daily for health status. At the experimental endpoint, rats were humanely euthanized via an intraperitoneal injection of sodium pentobarbital. Lung tissues were rapidly harvested, frozen in liquid nitrogen, and stored at −80 °C for further analysis.

### 2.3. Organ Coefficient Calculation

Lungs were isolated and stripped of extraneous fat and bronchial tissues. After weighing the tissue on an analytical balance, the lung coefficient was determined as the ratio of lung weight to terminal body weight: lung coefficient (%) = (lung weight/body weight) × 100%.

### 2.4. Cell Culture

Human lung adenocarcinoma epithelial cells (A549) were obtained from Servicebio Technology Co., Ltd. (Wuhan, China). The cells were cultured in DMEM supplemented with 10% fetal bovine serum (FBS) and 1% penicillin–streptomycin at 37 °C in a 5% CO_2_ incubator (Thermo Fisher Scientific, Waltham, MA, USA). To investigate the effects of MFN2, cells were randomly assigned to four groups: (1) control group; (2) coal dust group; (3) Ad-MFN2 + coal dust group; and (4) Ad-NC group. For adenoviral transfection, A549 cells were seeded in 6-well plates at a density of 2 × 10^5^ cells/well. Upon reaching 60–70% confluence, cells were transfected with Ad-MFN2 or Ad-NC (MOI = 50) (Vigene Biosciences, Jinan, China) in serum-free medium for 12 h. Subsequently, the medium was replaced with complete medium containing 200 μg/mL coal dust suspension for an additional 24 h of incubation.

### 2.5. Hematoxylin–Eosin (HE) and Masson Staining

Lung tissues were fixed in 4% paraformaldehyde (Solarbio, Beijing, China) for 24 h at room temperature, followed by dehydration through a graded ethanol series, clearing in xylene, and embedding in paraffin blocks. Sections with a thickness of 3.5 μm were obtained, deparaffinized, and rehydrated for histological assessment. For HE staining, sections were incubated with hematoxylin (Servicebio, Wuhan, China) for 5 min and eosin (Servicebio, Wuhan, China) for 2 min. Subsequently, the slides were dehydrated, cleared, and mounted. Histopathological changes were evaluated under an optical microscope (Leica, Wetzlar, Germany). Additionally, collagen deposition was visualized using Masson’s trichrome staining according to the manufacturer’s instructions (Servicebio, Wuhan, China).

### 2.6. CCK-8 Assay

The effect of coal dust on A549 cell viability was evaluated using the CCK-8 assay. Cells were seeded in 96-well flat-bottom plates at a density of 1 × 10^4^ cells/well and incubated at 37 °C for 24 h. Subsequently, the cells were treated with various concentrations of coal dust for an additional 24 h. Following treatment, 10 µL of CCK-8 reagent (Baoguang, Chongqing, China) was added to each well, and the plates were incubated in the dark for 2 h. The absorbance (optical density, OD) at 450 nm was measured using a microplate reader (Thermo Fisher Scientific, Waltham, MA, USA). Cell viability was calculated as a percentage relative to the control group. Based on these results, 200 μg/mL was determined as the optimal concentration for further studies.

### 2.7. Wound-Healing Assay

The wound-healing assay was used to determine cell migration motility. Straight lines were drawn on a 6-well plate with the help of a marker. After that, the seeding of 5 × 10^5^ cells was done on each well. After incubating for 24 h, two parallel scratches were vertically drawn across the plate with a 10 μL pipette tip perpendicular to that which was marked. The wells were then washed twice using PBS in order to clear off cellular debris, and cells in the scratched areas were scraped off carefully. Subsequently, serum-free medium was used. Another 24 h later, cell migration was studied and observed through a microscope and recorded.

### 2.8. Clone Formation Assay

The Petri dishes were seeded with 1 × 10^5^/mL cells. They were coated with a suspension of coal dust after 24 h and incubated further for 24 h in an incubator. Then the cells were digested and harvested, and 500 cells were placed in each fresh culture dish to proceed with the cultivation. The culture was ended once the clonal cell clusters were observed. PBS was then used twice to wash the cells and fixed with 4% paraformaldehyde for 20 min, stained with crystal violet (Solarbio, Beijing, China), photographed, and analyzed using ImageJ.

### 2.9. Determination of ROS, MDA, and SOD Levels

The intracellular levels of reactive oxygen species (ROS) were measured using the peroxide-sensitive fluorescent probe, DCFH-DA (following the instructions of Solarbio, Beijing, China). Malondialdehyde (MDA) and superoxide dismutase (SOD) were also measured using commercial kits, according to the guidelines (Jiancheng, Nanjing, China).

### 2.10. Transmission Electron Microscopy (TEM)

Fix a block of lung tissue of approximately 1 mm^3^ in 2.5% glutaraldehyde for at least 2 h and wash three times with PBS for a duration of 10 min for each cycle; then fix with 1% osmic acid for 2 h and wash with PBS three times, each time for 10 min. Tissues were dehydrated in gradients of 30%, 50%, 70%, and 90% ethanol for 10 min each, and twice in 100% ethanol for 10 min each. The tissues were embedded in epoxy resin, trimmed, and positioned. After localization, cutting into ultrathin sections, and staining, they were observed and photographed under a transmission electron microscope.

The cells were first processed and cultured as needed for the experiment. Discard the medium, then rinse the A549 cells twice with PBS, digest them, and transfer them to a centrifuge tube. After centrifugation at 1000 r/min for 5 min, remove the supernatant, and the cell pellet was resuspended in the same fixative for postfixation, dehydration, infiltration, and embedding as per the lung tissue sample protocol. Finally, observe the intracellular mitochondria-ER contact points in A549 cells under a transmission electron microscope (Hitachi Ltd., Tokyo, Japan) and capture images for analysis.

### 2.11. Western Blot (WB)

Western blot analysis assessed the protein levels of MFN2, MAMs, and apoptosis in rat tissues and A549 cells, which were sonicated for lysis. Proteins were separated via SDS-PAGE (Epizyme, Shanghai, China), transferred to PVDF membranes (Millipore, Billerica, MA, USA), blocked with 5% milk (BD Biosciences, San Jose, CA, USA) for 1 h at room temperature, then incubated overnight with primary antibodies at 4 °C. After washing, membranes were incubated with secondary antibodies for 1 h, washed again, and developed with chemiluminescent solution. Protein levels were normalized to GAPDH and detected using a chemiluminescent imaging system (Thermo Fisher Scientific, Waltham, MA, USA).

### 2.12. RT-qPCR

The TRIzol method (Invitrogen) extracted total RNA from A549 cells and rat lung tissues. RNA concentration was measured spectrophotometrically. The RNA was reverse transcribed into cDNA using a 20 µL reaction and a kit from TaKara Bio Inc. (Beijing, China). We measured gene expression levels using the 2^−ΔΔCT^ method, normalizing to GAPDH. The primer sequences for RT-qPCR are listed in Table S1.

### 2.13. Immunofluorescence Staining and Analysis

A549 cells on glass coverslips were divided into different groups. After fixation, permeabilisation, and blocking, the cells were incubated overnight at 4 °C with primary antibodies against IP3R and VDAC, then labeled with fluorescent secondary antibodies in the dark. Nuclei were stained with DAPI, and images were taken using confocal microscopy. Colocalization was assessed using Pearson’s correlation coefficient in ImageJ 1.54g software (Wayne Rasband, National Institutes of Health, Bethesda, MD, USA).

### 2.14. Mitochondrial Membrane Potential (∆ψm) Assay

Cells were seeded in 6-well plates (1 × 10^6^ cells/well) and cultured to 80–90% confluence. After treatment with serum-free DMEM or a ∆ψm inhibitor for 24 h, cells were incubated with JC-1 probe (Beyotime Biotechnology, Shanghai, China) at 37 °C for 20–30 min in the dark. After three PBS washes, red fluorescence (Ex/Em: 525/590 nm) was captured using an inverted fluorescence microscope (Nikon, Tokyo, Japan). Five random fields per well were recorded to assess mitochondrial function.

### 2.15. Intracellular Calcium Concentration Assay

Cells (1 × 10^6^ cells/well) were seeded in 6-well plates and treated with a calcium agonist for 24 h. Subsequently, cells were incubated with 2 mL (2–5 μmol/L) Rhod-2 AM (Beyotime Biotechnology, Shanghai, China) at 37 °C for 30–45 min. Following PBS washes to remove extracellular probes, red fluorescence (Ex/Em: 552/581 nm) was visualized under an inverted microscope. Five random fields per replicate were imaged to evaluate calcium distribution.

### 2.16. Cell Apoptosis Assay

Cells (1 × 10^4^ cells/well) in 96-well plates were treated for 24 h to induce apoptosis. Following PBS washes, cells were resuspended in 100 µL binding buffer and stained with 5 µL Annexin V-FITC and 5 µL PI (Beyotime Biotechnology, Shanghai, China) for 15 min at room temperature. Fluorescence intensities for FITC (Ex/Em: 488/525 nm) and PI (Ex/Em: 535/615 nm) were immediately measured using a microplate reader to calculate the total apoptotic rate.

### 2.17. Statistical Methods

The findings were expressed as mean and SD. GraphPad Prism 8 software was applied to perform statistical analysis (GraphPad Software, San Diego, CA, USA). ANOVAs were used to conduct statistical analysis on normally distributed values. The *p*-value < 0.05 was considered statistically significant.

## 3. Results

### 3.1. Coal Dust Exposure Induced Fibrosis in the Lung Tissues of SD Rats and A549 Cells

**Figure 1:**
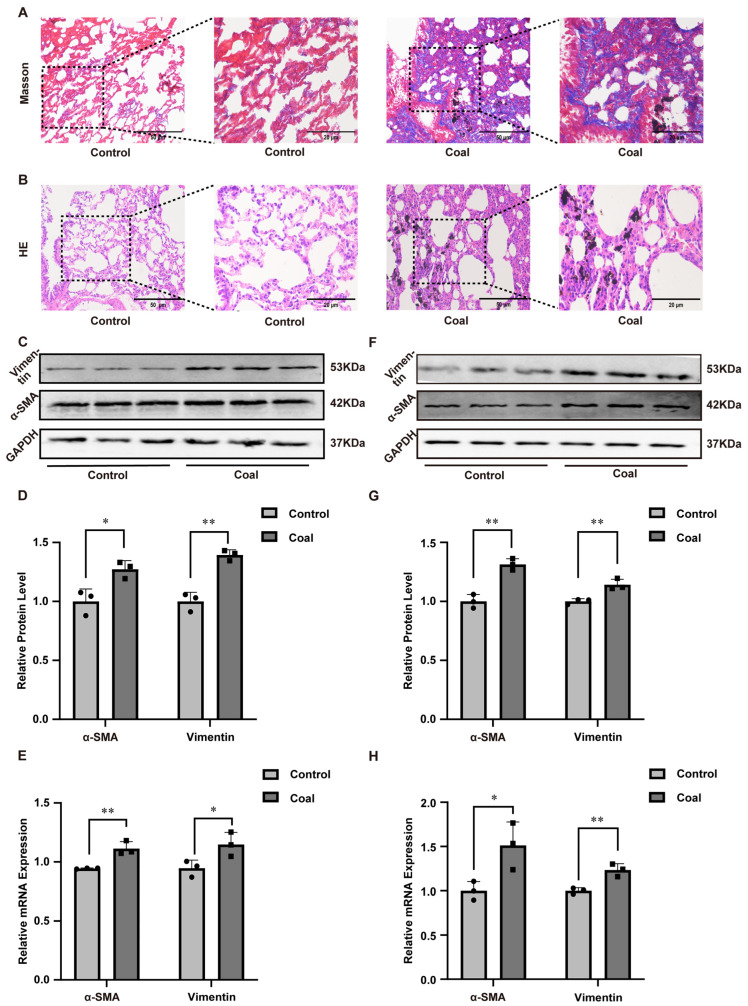
Coal dust exposure induced pulmonary fibrosis in vivo and in vitro. (**A**) Masson’s trichrome and (**B**) HE staining of rat lung tissues. (**C**,**D**) Western blot analysis of α-SMA and Vimentin protein levels in rat lung tissues. (**E**) RT-qPCR analysis of α-SMA and Vimentin mRNA levels in rat lung tissues. (**F**,**G**) Western blot analysis of α-SMA and Vimentin protein levels in A549 cells. (**H**) RT-qPCR analysis of α-SMA and Vimentin mRNA levels in A549 cells. Data are presented as mean ± SD relative to the untreated control. * *p* < 0.05, ** *p* < 0.01 vs. control, one-way ANOVA with Dunnett’s post-test. n ≥ 3.

Particle size analysis using scanning electron microscopy confirmed that all coal dust particles were less than 10 μm, which qualifies them as respirable dust (Figure S1A). A CWP model was then established in SD rats via intratracheal instillation of coal dust suspension. After modeling, rats exposed to coal dust exhibited listlessness, piloerection, tachypnea, orthopnea, and obvious pulmonary moist rales. Macroscopic observation of isolated lung tissues showed that lungs in the control group were smooth, pink, soft, and structurally normal. In contrast, lungs from coal dust-exposed rats appeared pale, hard, cystic, and displayed obvious coal particle deposition (Figure S1B). The lung organ coefficient was significantly higher in the dust-exposed group than in the control group, suggesting pathological swelling and injury induced by coal dust (Figure S1C). Masson staining revealed that collagen fibers in the control group were sparsely distributed around bronchi and vessels, whereas extensive collagen proliferation occurred in the alveolar septum and interstitial space in the dust-exposed group, with a markedly elevated positive area ratio (Figure 1A). HE staining further demonstrated severe alveolar structural destruction, alveolar septal thickening, inflammatory cell infiltration, and collagen deposition in coal dust-treated rats (Figure 1B). To further confirm the effect of coal dust on lung tissues, we employed Western blot and RT-qPCR to measure changes in protein and mRNA expression levels of Vimentin and α-SMA. As shown in Figure 1C–E, both protein and mRNA expression levels in the coal group increased, which indicated that dust exposure induced fibrosis in the lung tissues of SD rats.

Consistent results were verified in A549 cells. CCK-8 assay showed that coal dust reduced cell viability in a concentration-dependent manner (25, 50, 100, 200, 300, and 400 μg/mL), and 200 μg/mL was selected as the optimal concentration for subsequent experiments (Figure S2A). Wound-healing and colony formation assays demonstrated that coal dust exposure significantly inhibited the migration and proliferation of A549 cells (Figure S2B–E). In addition, coal dust exposure decreased superoxide dismutase (SOD) activity and adenosine triphosphate (ATP) levels, while increasing malondialdehyde (MDA) content in A549 cells (Figure S2F–H), indicating cellular toxicity and mitochondrial dysfunction. Furthermore, coal dust significantly upregulated the expression of Vimentin and α-SMA at both protein and mRNA levels in A549 cells (Figure 1F–H). Collectively, these results demonstrated that coal dust induced fibrotic responses in A549 cells.

### 3.2. Coal Dust Exposure Impaired Mitochondria-Associated Membranes (MAMs) Integrity

**Figure 2:**
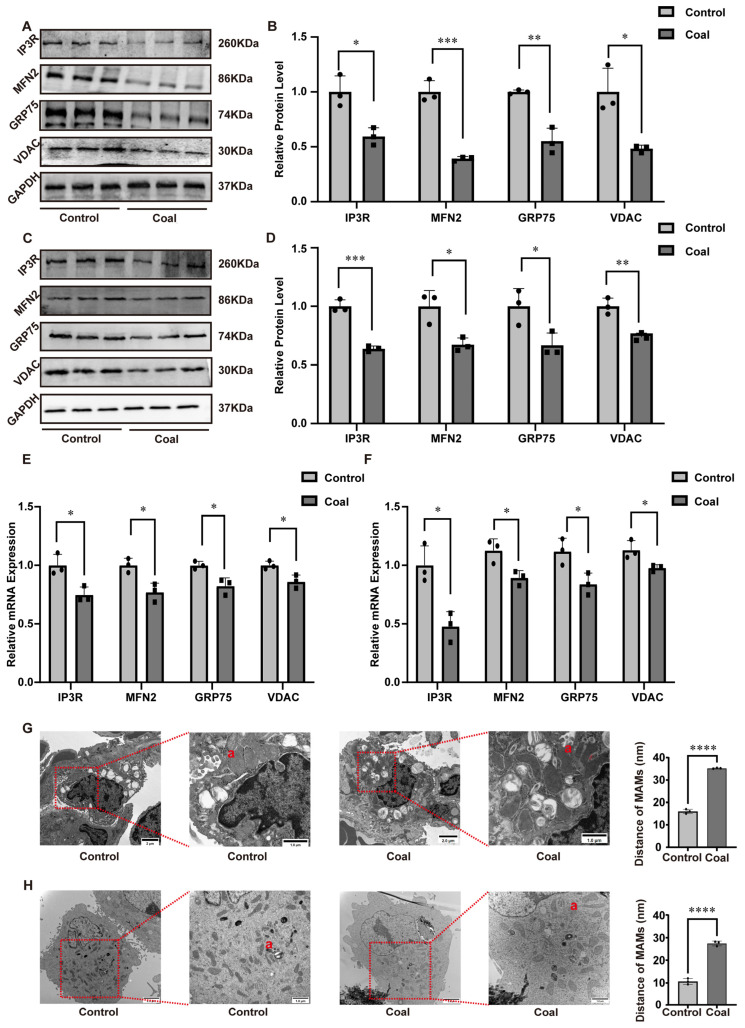
Impacts of coal dust exposure on the structure of MAMs in SD rats and A549 cells. (**A**,**B**) WB of related protein expression in rats’ lung tissues. (**C**,**D**) WB of related protein expression in A549 cells. (**E**) RT-qPCR of related mRNA levels in rats’ lung tissues. (**F**) RT-qPCR of related mRNA levels in A549 cells. (**G**) TEM and TEM image analysis of rats’ lung tissues. (**H**) TEM and TEM image analysis of A549 cells. Data are presented as mean ± SD relative to the untreated control. * *p* < 0.05, ** *p* < 0.01, *** *p* < 0.001, **** *p* < 0.0001 vs. control, one-way ANOVA with Dunnett’s post-test. n ≥ 3. a: The distance between mitochondria and endoplasmic reticulum.

Mitochondria–endoplasmic reticulum (ER) connections and functional crosstalk are primarily mediated by MAMs, whose structural integrity is pivotal for cellular signal transduction, material trafficking, and stress resilience. We first assessed molecular alterations within MAMs compartments by examining the protein abundance of core regulatory factors. WB analysis revealed a marked reduction in the levels of IP3R, MFN2, GRP75, and VDAC in both coal dust-exposed SD rat lung tissues and A549 cells, compared to respective controls (Figure 2A–D). Consistent with protein level changes, RT-qPCR assays confirmed a significant downregulation in the mRNA expression of these four key MAM components following dust exposure (Figure 2E–F). Furthermore, transmission electron microscopy (TEM) was employed to visualize MAM architecture in lung tissues and cells. In control groups, the ER remained tightly juxtaposed to mitochondria, displaying intact MAM structures with stable and distinct contact interfaces. In stark contrast, coal dust exposure induced severe MAM disruption, characterized by loose or fragmented connections between the ER and mitochondria. Quantitative image analysis demonstrated a significant increase in the inter-organellar distance alongside a reduction in both the contact length and the number of MAM contact sites (Figure 2G–H). Taken together, these findings demonstrated that coal dust exposure compromised the structural integrity of MAMs.

### 3.3. Coal Dust Exposure Disrupted MAM-Mediated Ca^2+^ Transport, Leading to Calcium Dyshomeostasis, Thereby Inducing Apoptosis

**Figure 3:**
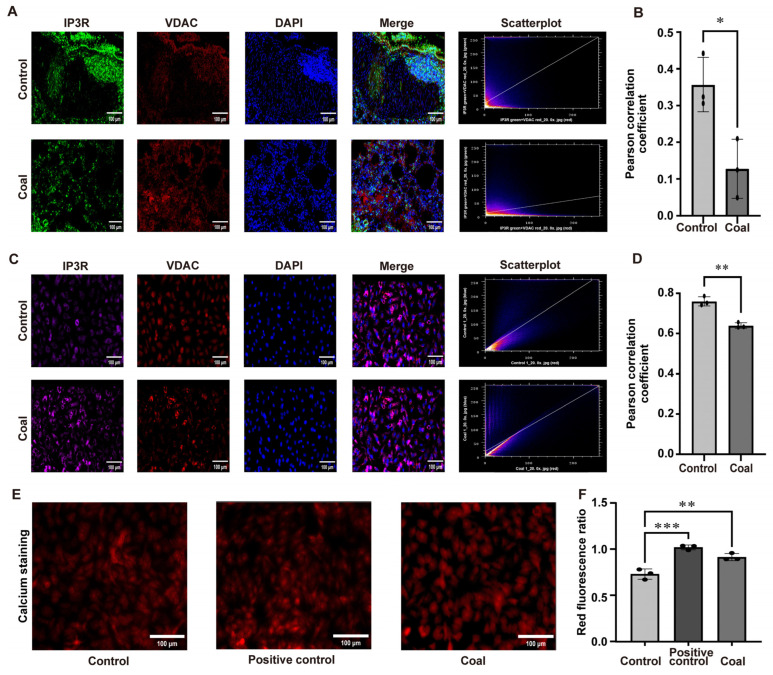
Effects of coal dust exposure on IP3R–VDAC colocalization at MAMs and mitochondrial calcium homeostasis. (**A**,**B**) Immunofluorescence co-staining of IP3R and VDAC in rats’ lung tissues. (**C**,**D**) Immunofluorescence co-staining of IP3R and VDAC in A549 cells. (**E**,**F**) Mitochondrial calcium staining and analysis. Data are presented as mean ± SD relative to the untreated control. * *p* < 0.05, ** *p* < 0.01, *** *p* < 0.001 vs. control, one-way ANOVA with Dunnett’s post-test. n ≥ 3.

To determine whether coal dust exposure interferes with the functional coupling between IP3R and VDAC at MAMs, we performed immunofluorescence (IF) co-localization analyses in lung tissues of SD rats (Figure 3A,B) and A549 cells (Figure 3C,D). In control groups, IF staining revealed a high degree of co-localization between IP3R (green) and VDAC (red), as evidenced by prominent yellow merged signals and a high Pearson correlation coefficient. In contrast, coal dust exposure markedly reduced the overlap of IP3R and VDAC signals, accompanied by a significant decrease in the Pearson correlation coefficient in both experimental systems. These observations indicated that coal dust impairs the physical interaction between IP3R and VDAC at MAMs, a key structural basis for efficient Ca^2+^ transport. We next assessed mitochondrial Ca^2+^ levels using a red fluorescent Ca^2+^ indicator (Figure 3E,F). The positive test group identified the validity of the assay. Compared with the control groups, coal dust exposure induced a substantial increase in mitochondrial red fluorescence intensity, indicative of mitochondrial Ca^2+^ overload. Taken together, these findings implied that coal dust disrupted MAM-mediated Ca^2+^ trafficking by destabilizing IP3R–VDAC coupling, thereby triggering mitochondrial calcium dyshomeostasis.

**Figure 4:**
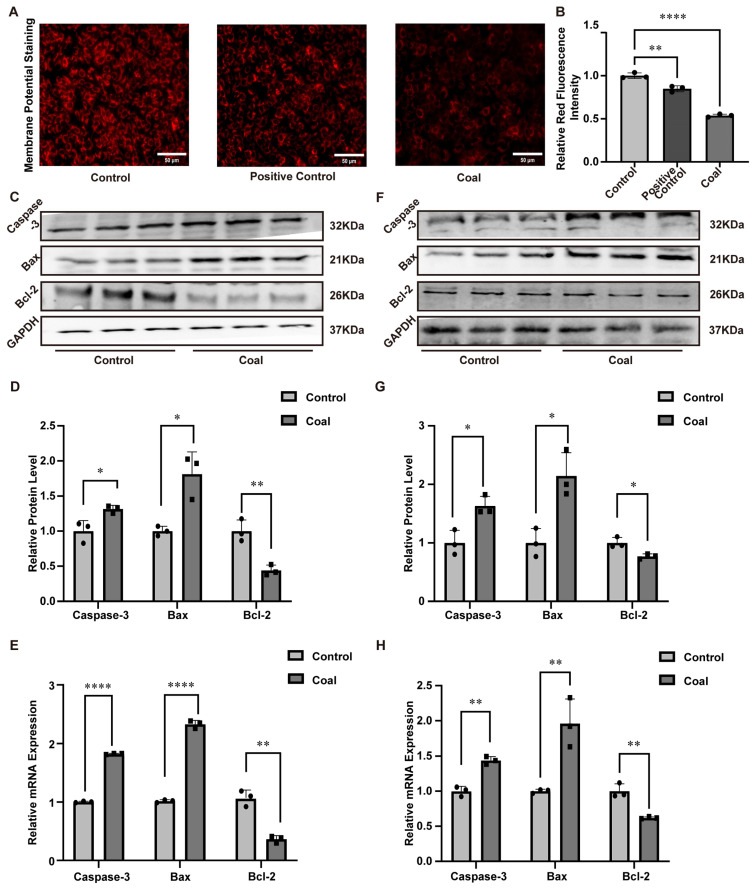
Impacts of coal dust exposure on apoptosis-related expression in SD rats and A549 cells. (**A**,**B**) Mitochondrial membrane potential fluorescence staining and quantification analysis. (**C**,**D**) Related protein expression in rats’ lung tissues. (**E**) Related mRNA levels in rats’ lung tissues. (**F**,**G**) Related protein expression in A549 cells. (**H**) Related mRNA levels in A549 cells. Data are presented as mean ± SD relative to the untreated control. * *p* < 0.05, ** *p* < 0.01, **** *p* < 0.0001 vs. control, one-way ANOVA with Dunnett’s post-test. n ≥ 3.

Mitochondrial membrane potential and apoptosis in A549 cells were evaluated by fluorescence staining. Control cells showed bright, homogeneous red fluorescence with intact mitochondrial structure and stable membrane potential. In contrast, the coal dust group exhibited markedly reduced red fluorescence, diffuse distribution, and even loss of fluorescence, indicating collapsed mitochondrial membrane potential and impaired mitochondrial function (Figure 4A). The same result was found through quantification analysis (Figure 4B). WB was carried out to identify the concentration of the most common apoptotic regulating proteins, Caspase-3, Bax, and Bcl-2, within the lung tissues of SD rats. The results showed that, compared with the control group, the protein levels of Caspase-3 and Bax were increased in the coal dust group, while the level of the anti-apoptotic protein Bcl-2 was decreased (Figure 4C,D). Meanwhile, RT-qPCR analysis of mRNA levels for these factors revealed consistent results (Figure 4E). We further determined the protein and mRNA levels of Caspase-3, Bax, and Bcl-2 in A549 cells, and the data were in accordance with those obtained from SD rats’ lung tissues (Figure 4F–H). Collectively, these findings indicated that coal dust exposure induced apoptosis.

### 3.4. MFN2 Overexpression Attenuated Coal Dust-Induced Pulmonary Fibrosis by Restoring MAMs’ Homeostasis and Inhibiting Apoptosis

**Figure 5:**
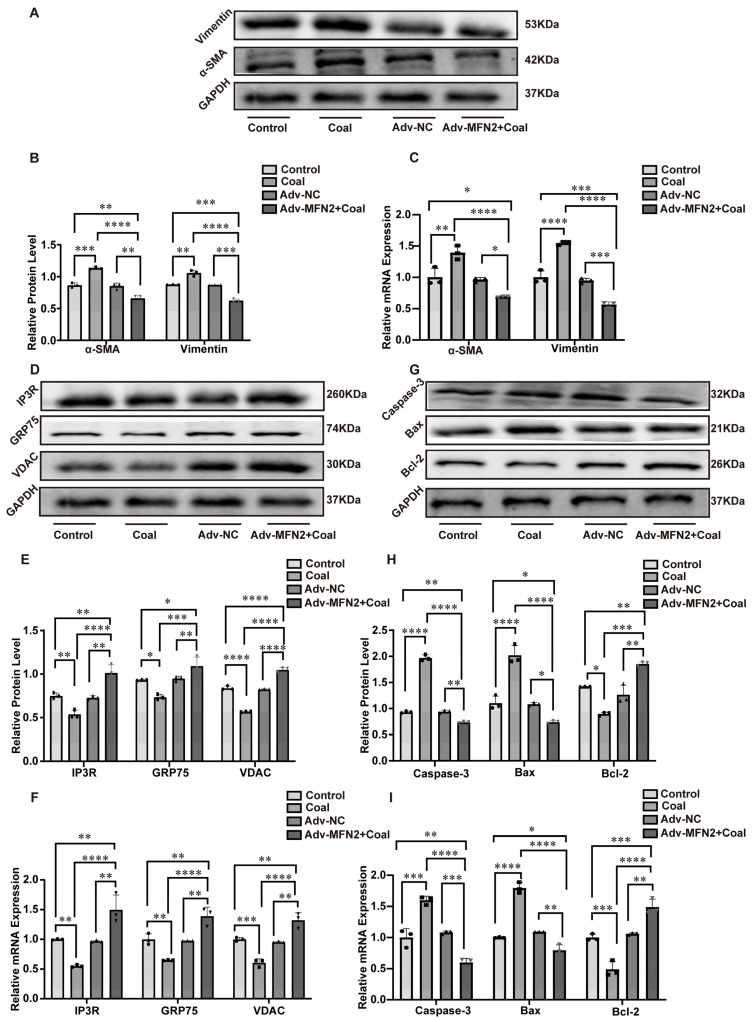
The relationship between MFN2 overexpression, pulmonary fibrosis, MAM structural integrity, and apoptosis. (**A**,**B**) WB images and quantitative analysis of α-SMA and Vimentin expression. (**C**) mRNA levels of α-SMA and Vimentin. (**D**,**E**) WB images and quantitative analysis of IP3R, GRP75, and VDAC expression. (**F**) mRNA levels of IP3R, GRP75, and VDAC. (**G**,**H**) WB images and quantitative analysis of Caspase-3, Bax, and Bcl-2 expression. (**I**) mRNA levels of Caspase-3, Bax, and Bcl-2. Data are presented as mean ± SD relative to the untreated control. * *p* < 0.05, ** *p* < 0.01, *** *p* < 0.001, **** *p* < 0.0001 vs. control, one-way ANOVA with Dunnett’s post-test. n ≥ 3.

To investigate the protective effect of MFN2 against coal dust-induced pulmonary fibrosis, A549 cells were transfected with MFN2-overexpressing adenovirus (Adv-MFN2) before coal dust treatment. WB and RT-qPCR confirmed successful overexpression of MFN2 (Figure S3A–C). MFN2 overexpression significantly inhibited coal dust-induced fibrotic responses, as evidenced by decreased protein and mRNA levels of α-SMA and Vimentin in the Adv-MFN2 + coal group (Figure 5A–C). Moreover, MFN2 overexpression restored the protein and mRNA expression of key MAMs components, including IP3R, GRP75, and VDAC (Figure 5D–F). In addition, overexpression of MFN2 reduced the levels of pro-apoptotic proteins Caspase-3 and Bax, while increasing the anti-apoptotic protein Bcl-2, with consistent changes at the mRNA level (Figure 5G–I). Collectively, these findings demonstrated that MFN2 overexpression alleviated coal dust-induced pulmonary fibrosis by restoring MAMs’ structural integrity and inhibiting apoptosis.

**Figure 6:**
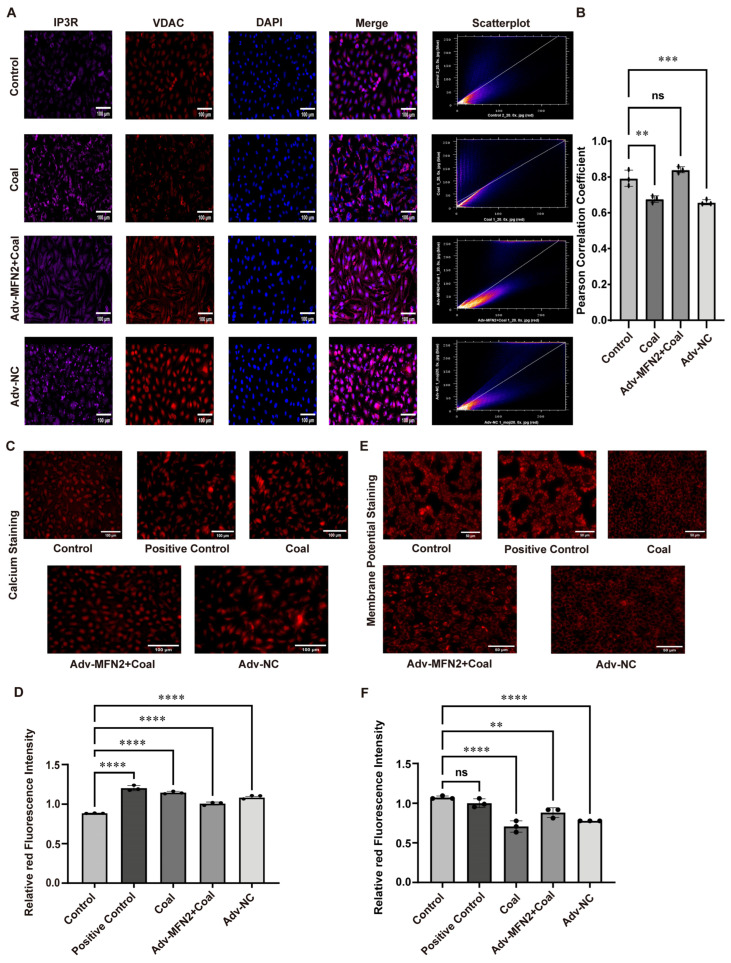
The impact of MFN2 overexpression. (**A**,**B**) Immunofluorescence co-staining of IP3R and VDAC and Pearson correlation coefficients. (**C**,**D**) Mitochondrial calcium staining and quantitative analysis. (**E**,**F**) Mitochondrial membrane potential staining and quantitative analysis. Data are presented as mean ± SD relative to the untreated control. ** *p* < 0.01, *** *p* < 0.001, **** *p* < 0.0001 vs. control, ns: not significant, one-way ANOVA with Dunnett’s post-test. n ≥ 3.

To further assess the effect of MFN2 overexpression, immunofluorescence co-staining of IP3R and VDAC was performed in A549 cells. In the control group, IP3R (purple) and VDAC (red) showed strong colocalization at MAMs with intense merged signals and a high Pearson correlation coefficient. Coal dust exposure markedly decreased IP3R–VDAC colocalization, suggesting damaged MAM structure. Importantly, MFN2 overexpression restored the colocalization of IP3R and VDAC in the Adv-MFN2 + coal group (Figure 6A,B). Mitochondrial calcium staining revealed that coal dust exposure markedly induced mitochondrial calcium overload, as evidenced by increased red fluorescence intensity. Importantly, MFN2 overexpression abrogated this effect, restoring mitochondrial calcium levels to those observed in the control group (Figure 6C,D).

Mitochondrial membrane potential staining showed that coal dust exposure markedly reduced red fluorescence intensity, indicating mitochondrial depolarization. This effect was significantly reversed in the Adv-MFN2 + coal group (Figure 6E,F). Flow cytometry using DCFH-DA demonstrated that coal dust exposure induced a prominent increase in intracellular ROS levels, leading to mitochondrial oxidative stress. In contrast, MFN2 overexpression significantly attenuated ROS accumulation (Figure S3D). Apoptosis assays further confirmed that MFN2 overexpression markedly alleviated coal dust-induced apoptosis (Figure S3E).

Taken together, these results revealed that MFN2 overexpression preserves MAMs’ structural integrity by restoring IP3R–VDAC colocalization, thereby mitigating mitochondrial calcium overload, restoring mitochondrial membrane potential, reducing ROS production, and inhibiting apoptosis in coal dust-exposed A549 cells.

## 4. Discussion

Coal workers’ pneumoconiosis (CWP) is a common occupational respiratory disease caused by sustained exposure to coal mine dust and is a public issue that affects the health of the global population. Even though the age-standardized incidence rate (ASIR) and disease burden of CWP declined between 1990 and 2019, the absolute number of incident cases is still high. Notably, no major decreases in the number of cases and ASIR have been reported in developing countries in the past years [2]. CWP is a progressive, irreversible disorder that has insidious early clinical features. With pulmonary fibrosis, patients develop a gradual cough, sputum, chest tightness, and progressive dyspnea; extreme cases may even progress to respiratory failure or cor pulmonale [20]. No effective therapies have so far been used to reverse the already established pulmonary fibrosis in large part because the pathogenic mechanisms involved are still incompletely elucidated.

Apoptosis is interconnected with fibrotic diseases in the form of a two-way feedback mechanism, which is especially prominent in pulmonary fibrosis [21]. The role of dysregulated apoptosis of lung parenchymal (particularly, alveolar epithelial) cells is one of the initiating trigger points in fibrogenesis. The release of damage-associated molecular patterns (DAMPs) and profibrotic factors by apoptotic cells directly induces the proliferation and differentiation of lung fibroblasts and thus accelerates the deposition of extracellular matrix (ECM) proteins (such as collagen). On the other hand, pulmonary fibrosis development brings about an imbalance in the pulmonary microenvironment that further increases parenchymal cell apoptosis by oxidative stress and inflammatory responses, creating a vicious cycle between fibrosis progression and apoptosis [22]. In accordance with this paradigm, our findings substantiated the fact that apoptosis interrelates well with fibrosis caused by coal dust. Coal dust exposure increased the apoptotic markers (caspase-3 and Bax) mRNA and protein expression and reduced the expression of the anti-apoptotic protein Bcl-2, which demonstrated the activation of the intrinsic apoptotic pathway. These observations are consistent with the established mechanism whereby calcium overload increases mitochondrial outer membrane permeability and triggers caspase activation [23]. Nevertheless, it is not well established that MFN2 suppressed CWP-linked fibrosis through regulating apoptotic signaling.

Over the last few years, a shift in perspective away from inflammation and oxidative stress towards a multi-pathway, network molecular control has been seen in studies on the pathogenesis of CWP. In this context, we have dwelled on the pivotal role of MFN2 and aimed to elucidate its effect in the concerted network that prevents fibrosis through the regulation of mitochondria-related endoplasmic reticulum membrane (MAMs) organization and apoptosis.

MAMs are vital signaling platforms that regulate intracellular Ca^2+^ homeostasis, signal transduction, stress responses, and apoptosis. Being functional hubs between the endoplasmic reticulum (ER) and mitochondria, the structural integrity and functional stability of MAMs are indispensable to ensuring normal cellular physiology, which is confirmed in numerous fibrotic diseases [24]. Under physiological circumstances, MAMs preserve structural integrity, guarantee the survival of cells through a calcium homeostasis control mechanism, and prevent the development of apoptotic programs. Structural damage or pathological deviations in the functioning of MAMs lead to a disequilibrium in calcium transportation between the ER and mitochondria, which causes mitochondrial failure and initiates the caspase-dependent intrinsic apoptotic pathway, resulting in aberrant cellular apoptosis [25]. This regulatory connection between structure/function, calcium homeostasis, and apoptosis in MAMs is especially essential in the pathophysiology of some diseases, like pulmonary fibrosis. In this study, we found that exposure to coal dust can significantly lower the expression levels of proteins and mRNA of the key components of MAMs, including MFN2, IP3R, GRP75, and VDAC. That was further confirmed by the results of TEM, where the coupling distance between mitochondria and endoplasmic reticulum has increased significantly. These results clarified the direct relationship between coal dust exposure and structural damage of MAMs.

Any impairment of the structural integrity of MAMs undermines their central role: the accurate regulation of the transfer of ER–mitochondrial Ca^2+^ [26]. It was observed that exposure to coal dust impaired the physical interaction between IP3R and VDAC at MAMs. When this spatial interaction is impaired, Ca^2+^ released at the ER is no longer efficiently translocated to mitochondria, leading to ER calcium overload and inadequate mitochondrial Ca^2+^ uptake. These changes initiate ER stress, inhibit mitochondrial energy metabolism, and eventually activate the intrinsic pathway of apoptosis, which stimulates cell apoptosis.

MFN2 is a key molecule required for MAMs formation and functional maintenance [27]. Beyond mediating mitochondrial fusion, MFN2 stabilizes ER–mitochondrial coupling by bridging the ER and outer mitochondrial membranes, thereby fine-tuning the efficiency of MAMs-dependent Ca^2+^ transport [28]. Accumulating evidence indicates that MFN2 critically regulates fibrotic processes in multiple organs, including the lung, liver, and kidney [29,30,31]. Its multifunctional roles are closely linked to the maintenance of cellular homeostasis. MFN2 is abundantly expressed in lung tissue and participates in lung injury repair and apoptotic balance, suggesting a deep involvement in the pathogenesis of pulmonary diseases. This is consistent with previous studies, which demonstrated that aberrant MFN2 expression correlates with impaired lung function [29]. In this study, we observed that coal dust exposure significantly downregulated MFN2 expression both in vitro and in vivo, which was closely associated with the progression of fibrosis and MAMs disruption. To further explore whether MFN2 plays a causal role in this process, we performed functional “rescue” experiments in vitro using adenoviral transduction. Our results demonstrated that MFN2 overexpression effectively reversed coal dust-induced MAMs structural damage and suppressed apoptosis in lung cells.

In summary, this study highlights the critical role of the MFN2–MAMs–Ca^2+^–apoptosis axis in the pathogenesis of CWP. Using a CWP model, we confirmed that coal dust exposure reduces MFN2 expression, increases collagen deposition, upregulates fibrotic markers (α-SMA, Vimentin), and induces oxidative stress, as evidenced by elevated ROS and MDA levels and reduced SOD activity. Concurrently, coal dust impaired MAMs’ structure, enhanced Caspase-3 activation, upregulated Bax, and downregulated Bcl-2. In contrast, adenovirus-mediated MFN2 overexpression significantly ameliorated these fibrotic and apoptotic abnormalities, inhibited fibrogenesis, and restored apoptosis near baseline levels. Nevertheless, several limitations in this study warrant cautious interpretation of the data. Although our findings establish a robust mechanistic foundation, the functional validation of MFN2 overexpression was confined to in vitro settings without corresponding gene-therapeutic interventions in our animal models. Furthermore, while the current experimental setup provides critical insights, it may not fully encapsulate the intricate chronic inflammatory landscape characteristic of human CWP. Crucially, as we strictly adhered to the 3Rs principles to prioritize animal welfare, the relatively modest sample size employed may inevitably constrain the statistical power and overall robustness of the in vivo observations. Such a limitation implies that the results could be sensitive to individual biological divergence. Consequently, future investigations utilizing larger cohorts and more diverse animal models (transgenic models or targeted gene delivery) are essential to solidify the MFN2–MAMs axis as a reliable clinical target.

## Data Availability

The original data presented in this study are included in the article/Supplementary Materials; further inquiries can be directed to the corresponding author.

## Supplementary Materials

The following supporting information can be downloaded at https://www.mdpi.com/article/10.3390/toxics14050391/s1. Figure S1: Establishment of the SD rat’s CWP models. (A) The particle size of coal dust. (B) Morphological changes in the rats’ lung tissues. (C) Lung organ coefficient in SD rats after exposure to coal dust. Lung organ coefficient = (lung tissue mass/animal body weight) × 100%. ** *p* < 0.01 vs. control, n ≥ 3. Figure S2: Coal dust cytotoxicity effects on A549 cells. (A) The effects of coal dust exposure on cell viability. (B,C) Migration ability of coal dust-exposed cells. (D,E) The clonogenic capacity of coal dust-exposed cells. (F) SOD activity of coal dust-exposed cells. (G) MDA content of coal dust-exposed cells. (H) ATP activity in A549 cells. * *p* < 0.05, ** *p* < 0.01, **** *p* < 0.0001 vs. control, n ≥ 3. Figure S3: Experiments related to MFN2 overexpression. (A,B) WB images and quantitative analysis of MFN2 protein expression. (C) RT-qPCR of MFN2 mRNA levels. (D) Intracellular ROS levels in each group. (E) Cellular apoptotic levels in each group. * *p* < 0.05, **** *p* < 0.0001 vs. control, ns: not significant, n ≥ 3. Table S1: Specific primers used for qRT-PCR.
