# Supplementary material for: MFN2 Overexpression Attenuates Coal Dust-Induced Pulmonary Fibrosis by Modulating MAMs Integrity and Cell Apoptosis
Source: Toxics. 2026 Apr 30;14(5):391. doi: 10.3390/toxics14050391 (PMC13211067; doi:10.3390/toxics14050391)
Supplement: Supplementary file 1 [file toxics-14-00391-s001.zip › toxics-4230919-supplementary.pdf]

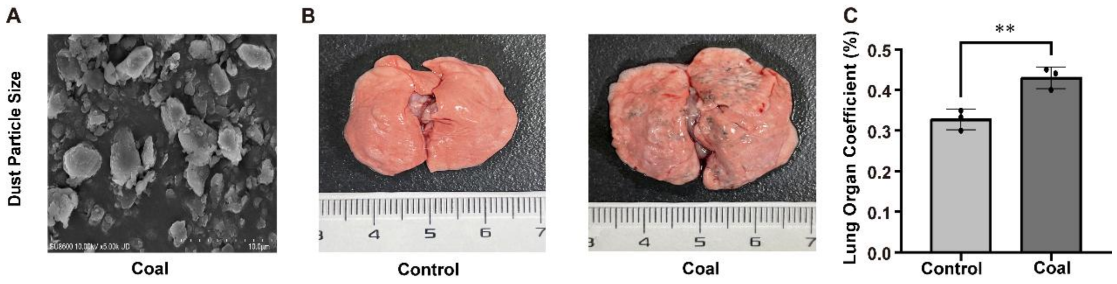

Figure. S1. Establishment of the SD rat's CWP models. (A) The particle size of coal dust. (B) Morphological changes in the rats' lung tissues. (C) Lung organ coefficient in SD rats after exposure to coal dust. Lung organ coefficient = (Lung Tissue Mass / Animal Body Weight) × 100%. **P <0.01 vs. control, n ≥ 3.

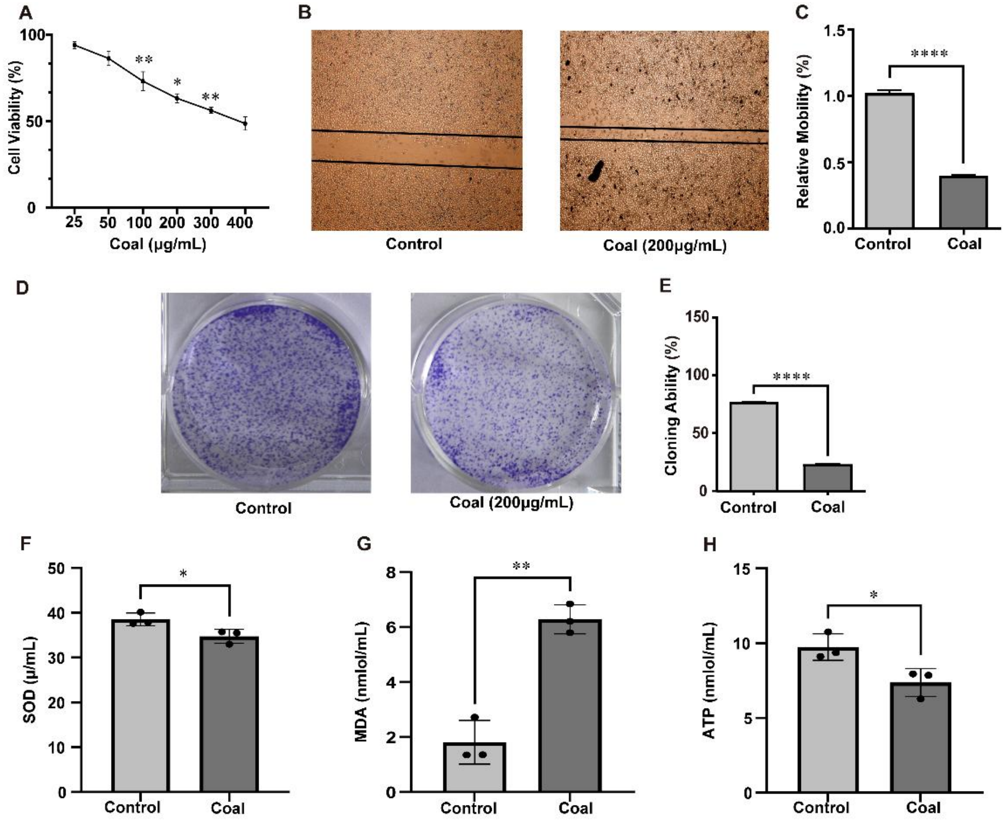

Figure. S2. Coal dust cytotoxicity effects on A549 cells. (A) The effects of coal dust exposure on cell viability. (B-C) Migration ability of coal dust-exposed cells. (D-E) The clonogenic capacity of coal dust-exposed cells. (F) SOD activity of coal dust-exposed cells. (G) MDA content of coal dust-exposed cells. (H) ATP activity in A549 cells. *P <0.05, **P <0.01, ****P<0.0001 vs. control, n ≥ 3.

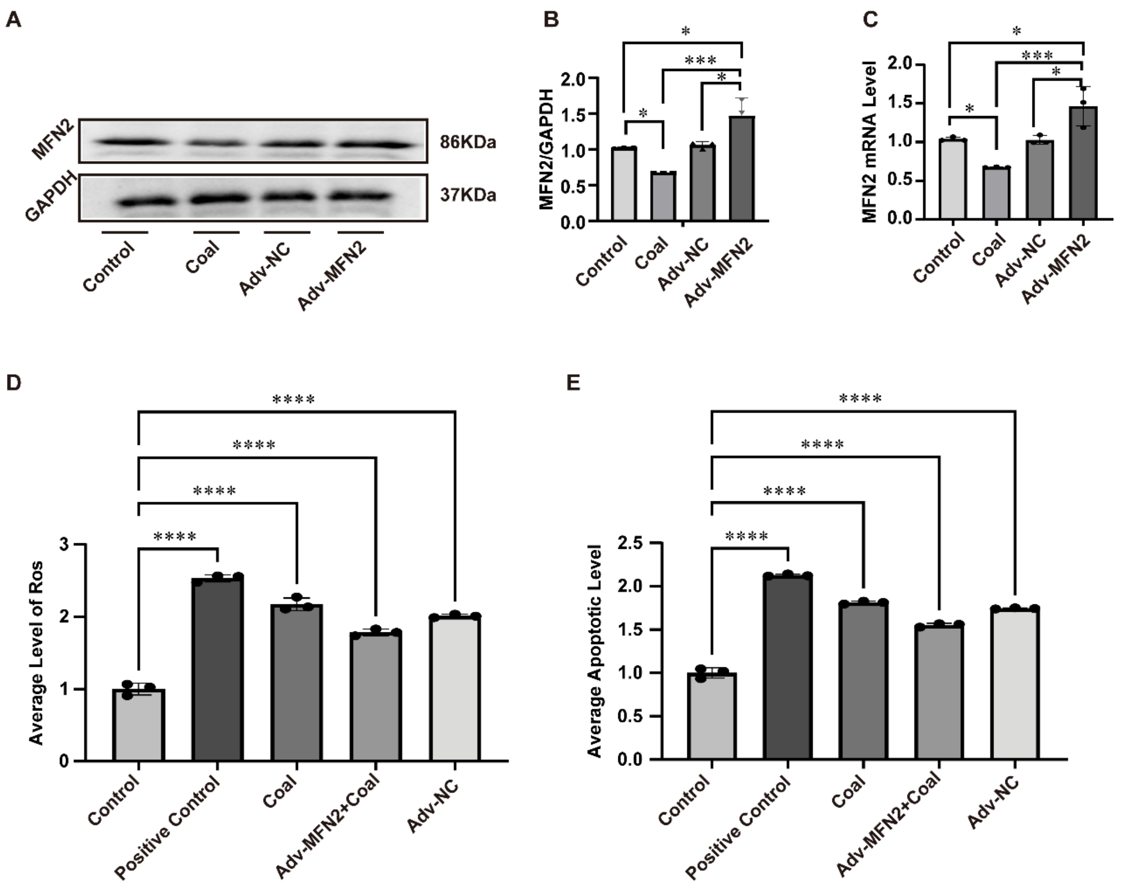

Figure. S3. Experiments related to MFN2 overexpression. (A-B) WB images and quantitative analysis of MFN2 protein expression. (C) RT-qPCR of MFN2 mRNA levels. (D) Intracellular ROS levels in each group. (E) Cellular apoptotic levels in each group. *P <0.05, ****P<0.0001 vs. control, ns: not significant, n ≥ 3. Table S 1: Specific primers used for qRT-PCR.

Table. S1. Specific primers used for qRT-PCR

	Gene	Forward	Reverse
**Rat**	Caspase-3	AGCACTGAAGACGCCATTG	GCCATGCGGTCGTAGAAAA
	Bcl-2	GGTGGGGTCATGTGTGTGG	CGGTTCAGGTACTCAGTCATCC
	Bax	GCGTCCACCAAGAAGCTGAG	CAGTTGAAGTTGCCGTCAGA
	α-sma	CCAGATCATTGCTCCTCCTG	CTTTGAAGGTGGTCTCGTGA
	Vimentin	CTGCCAAGAACCTCCAGGAG	ACTTCGCAGGTGAGTGACTG
	MFN2	GAAGAGATCAGGCGCCTCTC	AGCGGTCAGACATGTTTCGT
	VDAC	GATGGACCGAGTATGGGCTG	AAGGTCAGCTTCAGTCCACG
	IP3R	GGTAGAGACGGGGGAGAACT	GAGCACATCTCCTACTCCGC
	GRP75	TGATTGGAATTCCCCCAGCC	TCTGTTGCTCACGTCCTGTC
	GAPDH	GGCACAGTCAAGGCTGAGAATG	ATGGTGGTGAAGACGCCAGTA
**Cell**	Caspase-3	TgC ATA CTC CAC AgC ACC Tg	TCT gTT gCC ACC TTT Cgg TT
	Bcl-2	gAA CTg ggg gAg gAT TgT gg	CAT CCC AgC CTC CgT TAT CC
	Bax	AAg gTg CCg gAA CTg ATC Ag	gTC TTg gAT CCA gCC CAA CA

α-sma	GCCATGAAGATCAAGATCATCGTC	AGGTCCAGACGCAGGATGTT
Vimentin	ACCAGCCGCAGCCTCTACG	AGCGAGAAGTCCACCGAGTCC
MFN2	TCCCTCTGACGCCAGCCAAC	CCACACCACTCCTCCAACAACAAG
VDAC	ACAGAGTTTGGCGGCTCCATTTAC	GAAGCAGGCGTCAGGGTCAATC
IP3R	ACCAACGCTGACATCCTGATTGAG	ACACCTCTTCCTCGTCTTCTCCTG
GRP75	GGTGCTGGAGAATGCCGAAGG	TTCCAACAAGTCGCTCACCATCTG
GAPDH	CAg gAg gCA TTg CTg ATg AT	gAA ggC Tgg ggC TCA TTT
